# Cell‐based therapies have disease‐modifying effects on osteoarthritis in animal models: A systematic review by the ESSKA Orthobiologic Initiative. Part 3: Umbilical cord, placenta, and other sources for cell‐based injectable therapies

**DOI:** 10.1002/ksa.12472

**Published:** 2024-09-20

**Authors:** Yosef Sourugeon, Angelo Boffa, Carlotta Perucca Orfei, Laura de Girolamo, Jeremy Magalon, Mikel Sánchez, Thomas Tischer, Giuseppe Filardo, Lior Laver

**Affiliations:** ^1^ Division of Surgery, Orthopaedics Department Chaim Sheba Medical Centre Ramat Gan Israel; ^2^ Applied and Translational Research Center, IRCCS Istituto Ortopedico Rizzoli Bologna Italy; ^3^ Clinica Ortopedica e Traumatologica 2, IRCCS Istituto Ortopedico Rizzoli Bologna Italy; ^4^ Laboratorio di Biotecnologie Applicate all'Ortopedia, IRCCS Ospedale Galeazzi Sant'Ambrogio Milan Italy; ^5^ INSERM, NRA, C2VN Aix Marseille University Marseille France; ^6^ SAS Remedex Marseille France; ^7^ Cell Therapy Laboratory, Hôpital De La Conception, AP‐HM Marseille France; ^8^ Advanced Biological Therapy Unit, Hospital Vithas Vitoria Vitoria‑Gasteiz Spain; ^9^ Arthroscopic Surgery Unit, Hospital Vithas Vitoria Vitoria‐Gasteiz Spain; ^10^ Department of Orthopaedic and Trauma Surgery Malteser Waldkrankenhaus Erlangen Germany; ^11^ Department of Orthopaedic Surgery University of Rostock Rostock Germany; ^12^ Department of Surgery Service of Orthopaedics and Traumatology, EOC Lugano Switzerland; ^13^ Faculty of Biomedical Sciences Università Della Svizzera Italiana Lugano Switzerland; ^14^ Arthrosport Clinic Tel‑Aviv Israel; ^15^ Rappaport Faculty of Medicine, Technion University Hospital (IsraelInstitute of Technology) Haifa Israel; ^16^ Department of Orthopaedics Hillel Yaffe Medical Center (HYMC) Hadera Israel

**Keywords:** cartilage, mesenchymal stromal cells (MSCs), placenta, stem cells, synovial, umbilical cord

## Abstract

**Purpose:**

This systematic review aimed to investigate in animal models the presence of disease‐modifying effects driven by non‐bone marrow‐derived and non‐adipose‐derived products, with a particular focus on umbilical cord and placenta‐derived cell‐based therapies for the intra‐articular injective treatment of osteoarthritis (OA).

**Methods:**

A systematic review was performed on three electronic databases (PubMed, Web of Science and Embase) according to PRISMA guidelines. The results were synthesised to investigate disease‐modifying effects in preclinical animal studies comparing injectable umbilical cord, placenta, and other sources‐derived products with OA controls. The risk of bias was assessed using the SYRCLE tool.

**Results:**

A total of 80 studies were included (2314 animals). Cell therapies were most commonly obtained from the umbilical cord in 33 studies and placenta/amniotic tissue in 18. Cell products were xenogeneic in 61 studies and allogeneic in the remaining 19 studies. Overall, 25/27 (92.6%) of studies on umbilical cord‐derived products documented better results compared to OA controls in at least one of the following outcomes: macroscopic, histological and/or immunohistochemical findings, with 19/22 of studies (83.4%) show positive results at the cartilage level and 4/6 of studies (66.7%) at the synovial level. Placenta‐derived injectable products documented positive results in 13/16 (81.3%) of the studies, 12/15 (80.0%) at the cartilage level, and 2/4 (50.0%) at the synovial level, but 2/16 studies (12.5%) found overall worse results than OA controls. Other sources (embryonic, synovial, peripheral blood, dental pulp, cartilage, meniscus and muscle‐derived products) were investigated in fewer preclinical studies. The risk of bias was low in 42% of items, unclear in 49%, and high in 9% of items.

**Conclusion:**

Interest in cell‐based injectable therapies for OA treatment is soaring, particularly for alternatives to bone marrow and adipose tissue. While expanded umbilical cord mesenchymal stem cells reported auspicious disease‐modifying effects in preventing OA progression in animal models, placenta/amniotic tissue also reported deleterious effects on OA joints. Lower evidence has been found for other cellular sources such as embryonic, synovial, peripheral blood, dental‐pulp, cartilage, meniscus, and muscle‐derived products.

**Level of Evidence:**

Level II.

**Abbreviations**
ADAMTS5: A disintegrin and metalloproteinase with thrombospondin motifs 5
ASA: amniotic suspension allograft
BMAC: bone marrow aspirate concentrate
CTXII: C‐terminal telopeptide of type II collagen
EVs: extracellular vesicles
ICAM‐1: intercellular adhesion molecule 1
IHC: immunohistochemistry
IL‐1β: interleukin 1 beta
iPSCs: induced pluripotent stem cells
micro‐CT: micro‐computed tomography
MMP13: matrix metallopeptidase 13
MRI: magnetic resonance imaging
MSC: mesenchymal stromal cells
NO: nitric oxide
OA: osteoarthritis
PGE2: prostaglandin E2
PRISMA: Preferred Reporting Items for Systematic Reviews and Meta‐Analyses
PRP: platelet‐rich plasma
SVF: stromal vascular fraction
SYRCLE: Systematic Review Centre for Laboratory Animal Experimentation
TGFβ: transforming growth factor beta
TNFα: tumour necrosis factor alpha
UC‐MSC: umbilical cord mesenchymal stromal cells

## INTRODUCTION

Osteoarthritis (OA) is the most common form of joint degenerative disease and one of the major causes of pain and disability in older adults, affecting around 3.6% of the population globally with a heavy burden on healthcare systems [10, 32]. Current non‐operative treatment paradigms are focused on symptom relief, with benefits often of limited duration, rather than providing disease‐modifying effects and changing the OA course. The limitations of these treatments in addressing altered tissue homoeostasis in OA have led to an increasing interest in orthobiologic strategies. Relying on their regenerative and immuno‐modulating properties through cellular mechanisms or blood‐derived proteins, they have been suggested to have the potential to restore homoeostasis in many tissues, including cartilage and overall joint tissues [12]. Recently, the European Society of Sports Traumatology, Knee Surgery and Arthroscopy (ESSKA) Orthobiologic Initiative (ORBIT) underlined OA disease‐modification properties in animal models for orthobiologic agents such as platelet‐rich plasma (PRP) [9] and mesenchymal stromal cells (MSCs) from adipose tissue [56] and bone marrow [8].

Given their therapeutic properties, MSCs have been suggested to be suitable candidates for treating damaged tissues. These cells are found in most vascularises tissues, especially in adipose tissue and bone marrow, although other sources have also been suggested. The current review will focus on other tissue sources for MSCs, including foetal annexes (i.e., placenta and amnion), synovial tissue, blood‐derived, muscle‐derived, and induced pluripotent stem cells (iPSCs). Compared to bone marrow and adipose tissue, these sources are more challenging to obtain due to procedural complexity, regulatory and ethical issues, lower MSC yield, or the need for lab manipulation [3]. Despite the source, abundant literature showed that MSCs exert an anti‐inflammatory effect on chondrocytes and synoviocytes and modulate the immune system response towards an anti‐inflammatory phenotype. In this light, the MSCs from various sources may affect the cartilage tissue of OA patients in two ways: (1) counteract the inflammatory processes in the cartilage tissue; (2) induce a chondrogenic response in the cartilage tissue. The culminating effect of these two processes may lead to a disease‐modifying effect on the affected joint [55, 70]. Transcriptional and epigenetic analysis of different MSC populations has been shown to have striking similarities [7]. However, not all MSCs are the same, as the cells' efficacy might also be source‐dependent [65]. The difference lies in the expressed growth factors or active pathways between the various MSC sources, which may affect the chondrogenic potential of each cell source and the required conditions required for such a process, thus underlining the importance of exploring the potential of the different sources when applied to address OA processes [7].

This systematic review, Part 3 of a series of publications by the ESSKA ORBIT on cell‐based products for OA treatment, aimed to investigate in animal models the presence of disease‐modifying effects driven by non‐bone marrow‐derived and non‐adipose‐derived products, with a particular focus on umbilical cord and placenta‐derived cell‐based therapies for the intra‐articular injective treatment of OA. This study hypothesises that cell‐based injectable therapies derived from umbilical cord and placental tissues will exhibit significant disease‐modifying effects on osteoarthritis in animal models.

## MATERIALS AND METHODS

### Search strategy and article selection

A systematic review of the literature was conducted on three electronic databases (PubMed, Web of Science, and Embase) according to the Preferred Reporting Items for Systematic Reviews and Meta‐Analyses (PRISMA) guidelines. The methodology of this systematic review, divided into three articles (according to different MSCs sources), was already reported in previous publications [8, 56]. Two authors (CP and YS) conducted the screening process and analysis, and a third author (AB) was involved in resolving any discrepancies. Preclinical studies focusing on the intra‐articular use of MSCs to address joints affected by OA were included based on the following inclusion criteria: Animal studies, articles written in English, purely injective treatments for cartilage degeneration and OA. Exclusion criteria were: In vitro or clinical studies, congress abstracts, reviews, articles written in other languages than English, studies on joint diseases different from OA, studies analysing associated surgery, studies on the use of MSC secretome/extracellular vesicles, and studies reporting the use of MSCs without a control group or the combined use of MSCs with another product without analysing the specific contribution of MSCs application.

**Figure 1:**
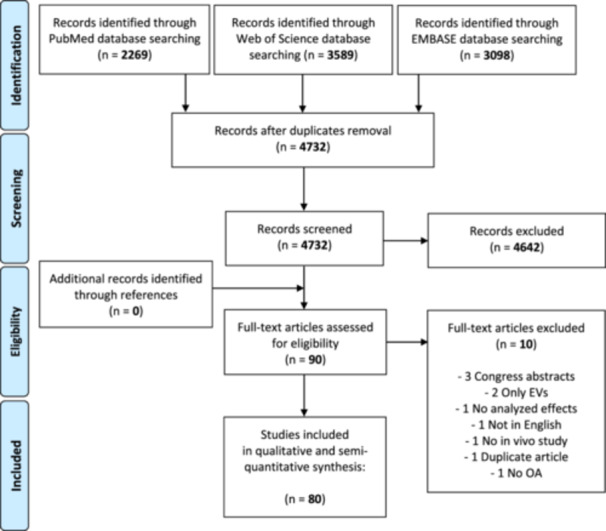
PRISMA flowchart of the study selection process. EVs, extracellular vesicles, OA, osteoarthritis.

While the first two articles of this ORBIT series analysed the disease‐modifying effects of adipose tissue‐derived products and bone marrow‐derived products, respectively [8, 56], the current manuscript focuses on other remaining MSC sources. The flowchart reported in Figure 1 graphically describes the systematic review process updated on 1 July 2023 for this third party using the same string: (MSC OR mesenchymal cell OR stem cell OR stromal cell OR progenitor cell OR bone marrow concentrate OR bone marrow aspirate concentrate OR BMAC OR micro‐fra* adipose tissue OR microfra* adipose tissue OR stromal vascular fraction OR SVF OR amniotic suspension allograft OR ASA OR placenta* OR umbilical cord OR amnio*) AND (osteoarthritis).

### Data extraction and quality assessment

Relevant data were extracted from article texts, tables, and figures and then collected in a database to be analysed for the purpose of the present study. In detail, the following data were collected: authors, journal, year of publication, number and type of evaluated animals, joint involved, OA model, type of treatment, follow‐up, results, and cell product characteristics, including source, origin, MSC count, additional procedures, processing modality (expanded versus “point‐of‐care”), and injective protocol. A synthesis of the obtained results was performed, analysing the clinical and imaging findings as well as the disease‐modifying effects on the OA process of the different preparations. The effects provided by the cell‐based injectable therapies were considered positive from a disease‐modifying perspective when the study reported better objective results (with a statistical significance) for cell therapies compared to OA controls in at least one of the following outcomes: macroscopic, histological, and/or immunohistochemical findings. In particular, the analysis was based on comparing the experimental groups versus the respective controls (vehicle injection or no treatment). The risk of bias assessment of the included studies was performed using the Systematic Review Centre for Laboratory Animal Experimentation (SYRCLE)'s tool [33].

## RESULTS

### Study selection and analysis

**Figure 2:**
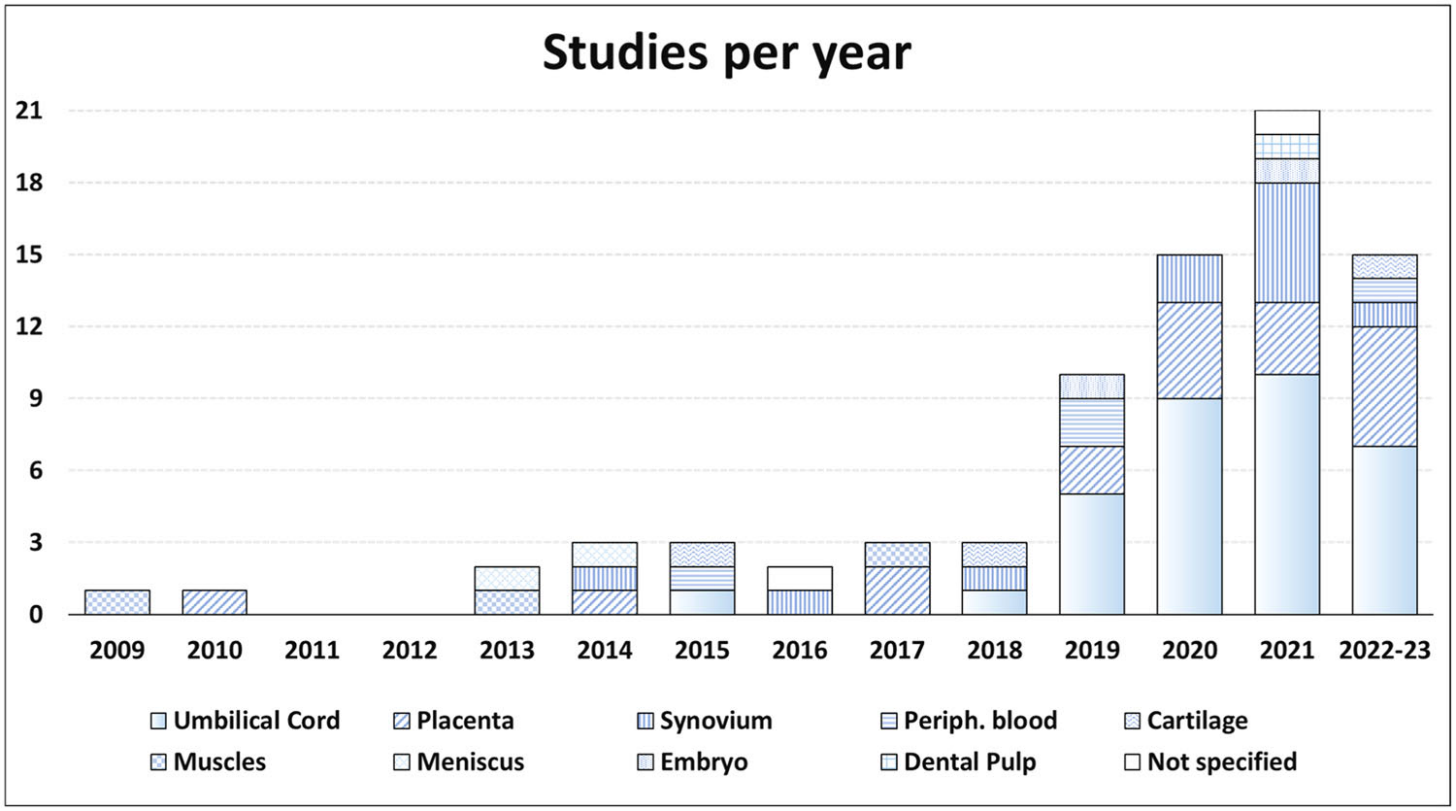
Animal studies over the years on intra‐articular injections of cell therapies different to adipose tissue and bone marrow to address OA. X axis, year of publication; Y axis, number of published articles.

Out of 4732 titles initially identified, 80 studies met the inclusion criteria after screening (Figure 1). These studies, which showed a significant increase in publications after 2018, focused primarily on cell therapies derived from umbilical cord and placental tissues. Small animal models, mainly rodents and rabbits, were most commonly used, with most studies evaluating the effects of expanded MSCs. The diversity in cell sources and the distribution of studies across different animal models are further detailed in Figures 1 and 2.

Sixty‐seven studies evaluated the effects on small animal OA models (rodents: 55; rabbits: 12) while 13 studies focused on large animals (dogs: 4: horses: 3; sheep: 3; goats: 1; monkeys: 1; and pigs: 1). The number of assessed animals were reported in 75 studies, with a total of 2314 animals: 1831 rodents, 208 rabbits, 129 dogs, 59 sheep, 42 horses, 27 goats, 10 pigs, and 8 monkeys. The treated joint was the knee in most studies (74), the elbow in two studies, the metacarpophalangeal in two studies, the fetlock joint in one study, and the temporomandibular joint in one study. The OA model was surgically induced in 45 studies (mostly through ligament transection and/or meniscectomy), chemically induced in 29 studies (through the injection of pro‐inflammatory or chondrotoxic products), or naturally occurring in six studies (veterinary clinical studies). Expanded MSCs were evaluated in 69 studies, while “point‐of‐care” products were analysed in 11 studies (all small animal studies). For expanded MSCs, the injected dose ranged from 3.0 × 10^4^ to 1.8 × 10^8^ cells for small animals and from 1.0 × 10^6^ to 5.0 × 10^7^ cells for large ones. Cell products were xenogeneic in 61 studies and allogeneic in the remaining 19 studies. The amount of the injected volume ranged from 25.0 μL to 4.0 mL in small animals and from 0.1 mL to 2.0 mL in large animals. The injective protocol consisted of a single injection in 52 studies, multiple injections (from 2 to 14) in 19 studies, seven studies compared different injective protocols, while two studies did not describe the injection schedule. The follow‐up period of the included studies ranged from 2 weeks to 12 months following OA induction. Further details on the characteristics of the included studies are reported in Supporting Information: Table 1, while the following paragraphs describe the disease‐modifying effects of cell therapies based on the cell source.

### Umbilical cord‐derived injectable products

**Figure 3:**
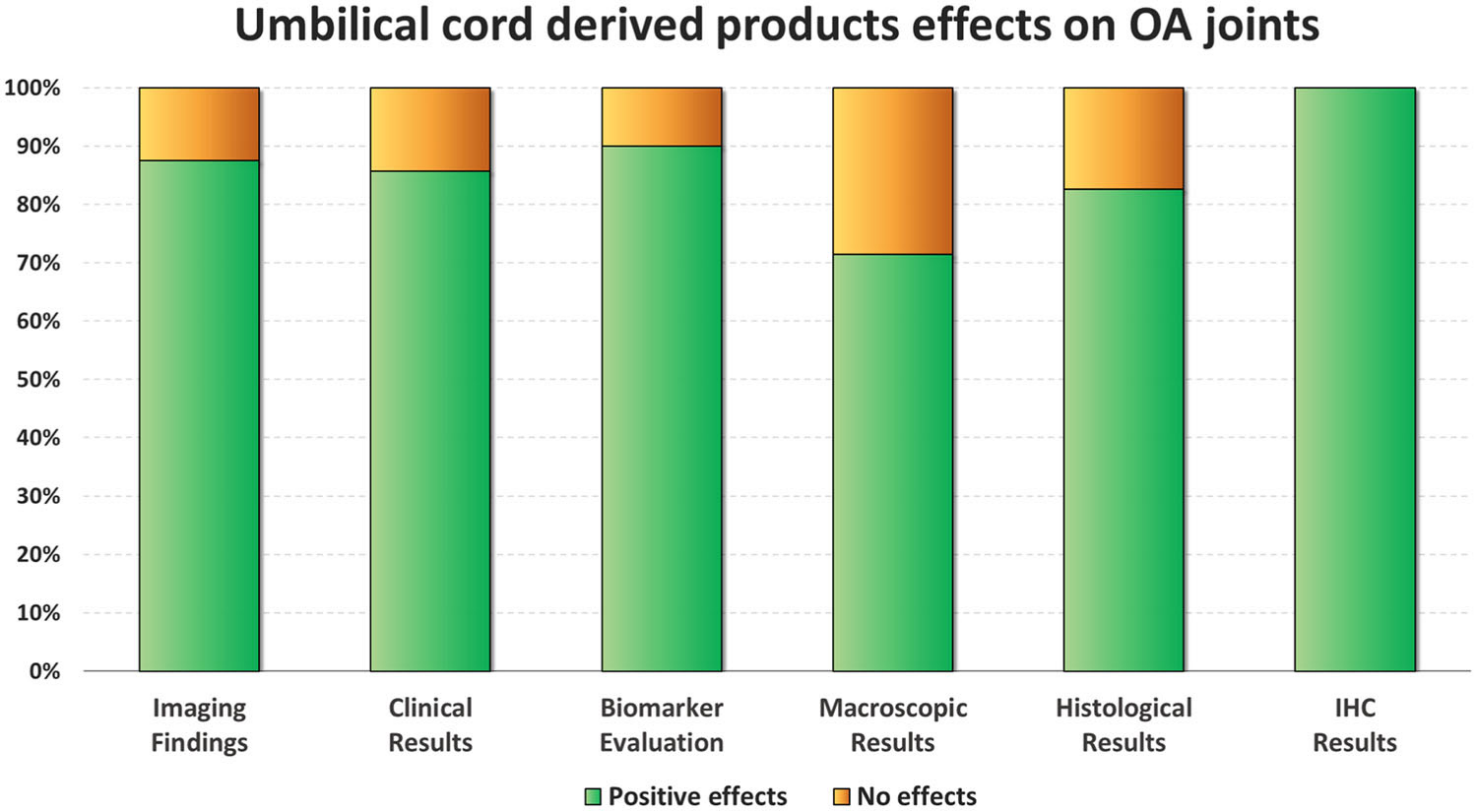
Disease‐modifying effects on OA joints induced by umbilical cord‐derived products. The bar chart shows the percentage of studies that met the specific effects. Positive effects (green) vs. no effects (orange) in imaging findings (*n* = 8), clinical results (*n* = 7), biomarker evaluation (*n* = 10), macroscopic results (*n* = 7), histological results (*n* = 23), and immunohistochemical results (*n* = 12). IHC, immunohistochemistry; OA, osteoarthritis.

A total of 33 studies analysed the injective use of expanded umbilical cord MSCs (UC‐MSCs) in animal OA joints. Out of these, 27 studies investigated their disease‐modifying effects concerning OA controls (vehicle injections or untreated joints). Overall, 25/27 studies (92.6%) documented better results compared to OA controls in at least one of the following outcomes: macroscopic, histological, and/or immunohistochemical findings, while the remaining 2/27 studies (7.4%) reported no improvement from the injective treatment. In detail, overall better results in favour to UC‐MSCs treatment were documented in 5/7 studies (71.4%) for macroscopic evaluations (gross morphological scores), in 19/23 studies (82.6%) for histological evaluations, and 12/12 studies (100.0%) for immunohistochemical analyses. A summary of UC‐MSCs effects on OA joints is reported in Figure 3, while more specific details on their effects are reported in Table 1.

**Table 1:** Disease‐modifying (DM) effects of umbilical cord‐derived injectable products.

	Results compared to OA controls	Details
DM effects at cartilage level	19/22 studies (83.4%) → Positive results	Better articular surface, less cartilage fibrillation, higher chondrocyte count, upregulation of aggrecan and type 2 collagen, and lower staining of IL‐1β, TNFα, MMP13, and ADAMTS5 compared to OA controls [6, 27, 37, 51, 68, 71, 84].
DM effects at synovial level	4/6 studies (66.7%) → Positive results	Lower inflammatory cell infiltration and hyperplasia compared to OA controls [15, 34, 71].
Effects on OA biomarkers	9/10 studies (90.0%) → Positive results	Higher serum levels of IL‐10 and synovial fluid levels of TGFβ. Lower serum levels of IL‐6, TNFα, and NO, and synovial fluid levels of IL‐1β, IL‐6, TNFα, and MMP13 [21, 45, 83, 84].
Clinical effects	6/7 studies (85.7%) → Positive results	Higher peak vertical force, better thermal withdrawal latency, better paw withdrawal threshold, and lower lameness [38, 39, 80, 85].
Imaging findings	7/8 studies (87.5%) → Positive results	Micro‐CT analysis (positive results in 5/5 studies): lower grade of OA change, larger joint space, and higher trabecular thickness and bone volume fraction [14, 15, 34, 53, 77]. Radiographic evaluation (positive results in 2/3 studies): less osteophyte formation and joint narrowing after UC‐MSCs treatment [4, 38, 77]. MRI (positive results in 1/2 studies): better cartilage thickness and volume [4, 83].Ultrasonographic evaluation: one study not reporting benefits [4].

Abbreviations: ADAMTS5, a disintegrin and metalloproteinase with thrombospondin motifs 5; IL, interleukin; Micro‐CT, micro‐computed tomography; MMP13, matrix metallopeptidase 13; MRI, magnetic resonance imaging; NO, nitric oxide; OA, osteoarthritis; TGFβ, transforming growth factor beta; TNF‐α, tumour necrosis factor‐α; UC‐MSC, umbilical cord mesenchymal stromal cells.

### Placental tissue‐derived injectable products

**Figure 4:**
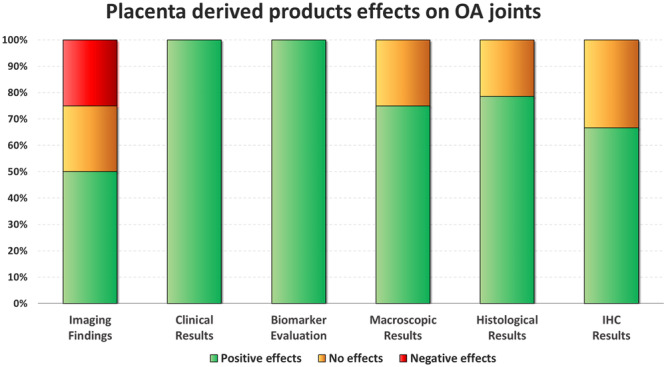
Disease‐modifying effects on OA joints induced by placental tissue‐derived injectable products. The bar chart shows the percentage of studies that met the specific effects. Positive effects (green) vs. no effects (orange) vs. negative effects (red) in imaging findings (*n* = 4), clinical results (*n* = 6), biomarker evaluation (*n* = 10), macroscopic results (*n* = 4), histological results (*n* = 14) and immunohistochemical results (*n* = 3). IHC, immunohistochemistry; OA, osteoarthritis.

The effects of placental tissue‐derived injectable products on animal OA models were evaluated in 18 studies: 11 studies focused on “point of care” products, while seven studies focused on expanded MSCs. Out of these 18 studies, 16 investigated the disease‐modifying effects of these products with respect to OA controls. Overall, 13/16 of studies (81.3%) documented better results compared to OA controls in at least one of the following outcomes: macroscopic, histological, and/or immunohistochemical findings, one study (6.3%) reported no improvement from the injective treatment, while 2/16 studies (12.5%) found worse results than OA controls. In detail, overall better results were documented in 3/4 studies (75.0%) for macroscopic evaluations (gross morphological scores), in 11/14 studies (78.6%) for histological evaluations, and in 2/3 studies (66.7%) for immunohistochemical analyses. A summary of placental tissue‐derived injectable products effects on OA joints is reported in Figure 4, while more specific details on their effects are reported in Table 2.

**Table 2:** Disease‐modifying (DM) effects of placental tissue‐derived injectable products.

	Results compared to OA controls	Details
DM effects at cartilage level	12/15 studies (80.0%) → Positive results	Lower cartilage degeneration and articular fibrillation, higher staining of type 2 collagen and Sox‐9, and lower staining of MMP13, ADAMT4, and ADAMT5 [23, 26, 48, 66].
DM effects at synovial level	2/4 studies (50.0%) → Positive results	Better synovitis scores [47, 77].
Effects on OA biomarkers	6/6 studies (100.0%) → Positive results	Higher serum levels of IL‐4, IL‐10, and TIMP‐1, and synovial fluid levels of IL‐2 and IL‐10. Lower serum levels of IL‐1β, IL‐6, TNFα, ICAM‐1, leptin, and selectin, and synovial fluid levels of IL‐1β, IL‐6, CTXII, TNFα, and PGE2 [5, 41, 42, 73, 76].
Clinical effects	4/4 studies (100.0%) → Positive results	Better clinical outcomes in terms of pain threshold levels, joint swelling, and weight‐bearing differences [5, 26, 41, 42].
Imaging findings	2/4 studies (50.0%) → Positive results	Micro‐CT analysis (positive results in 2/4 studies): greater articular cartilage thickness and volume. Negative results in one study reporting higher cartilage surface roughness and osteophyte cartilage thickness [41, 59, 63, 78].

Abbreviations: ADAMTS, a disintegrin and metalloproteinase with thrombospondin motifs; CTXII, C‐terminal telopeptide of type II collagen; ICAM‐1, intercellular adhesion molecule 1; IL, interleukin; Micro‐CT, micro‐computed tomography; MMP13, matrix metallopeptidase 13; OA, osteoarthritis; PGE2, prostaglandin E2; TNF‐α, tumour necrosis factor‐α.

### Other cell sources

Disease‐modifying effects induced by cell therapies obtained from the remaining sources have been analysed in less than 10 studies per source. Therefore, they are all described in this paragraph.

Eleven studies evaluated the injective use of expanded synovial fluid/membrane MSCs in animal OA joints, of which nine reported objective outcomes. In detail, 8/9 studies (88.9%) documented better results compared to OA controls in at least one of the following outcomes: macroscopic, histological, and/or immunohistochemical findings, while one study (11.1%) reported no improvement. Disease‐modifying effects were documented at both cartilage and synovial membrane levels. Moreover, three studies conducted a biomarker evaluation, all reporting positive changes in inflammatory and catabolic markers compared to OA controls [22, 58, 75]. Three studies also evaluated imaging outcomes, finding positive results after the injections in two studies (MRI and radiograph analyses) and no benefits in one study (MRI analysis) [31, 40, 81]. Finally, no studies assessed clinical outcomes after synovial fluid/membrane‐derived cell products.

Four studies evaluated the injective use of expanded peripheral blood MSCs in animal OA joints, of which only two reported objective outcomes: both studies documented better results compared to OA controls in terms of macroscopic, histological, and immunohistochemical findings [11, 44]. The same two studies documented positive changes in terms of serum and synovial fluid biomarker levels [11, 44]. Regarding imaging evaluations, one study reported positive findings at the subchondral level at micro‐CT analysis, while one study did not find any benefits at radiographic evaluation [11, 44]. Regarding clinical assessment, one study reported better clinical scores in OA animals treated with peripheral blood MSCs injections, while one study did not find any clinical benefits [11, 17].

Three studies evaluated the injective use of expanded cartilage‐derived MSCs in animal OA joints. All these studies documented better results compared to OA controls in terms of histological and immunohistochemical findings at cartilage levels [18, 24, 79]. One of these studies also detected positive changes in the biomarkers profile compared to OA controls [24].

Three studies evaluated the injective use of expanded muscle‐derived MSCs in animal OA joints. Two of these studies documented better results compared to OA controls in terms of histological and immunohistochemical findings at the cartilage level, while the third study did not find any benefit [13, 49, 50]. One of these studies also detected positive changes in the biomarker profile compared to OA controls [13].

The remaining studies, focusing on expanded MSCs derived from meniscus (two studies), embryonic tissue (one study), epidermidis (one study), and dental pulp (one study), also reported overall positive disease‐modifying effects. Supporting Information: Table 1 provides further details on the characteristics of these studies.

### Risk of bias assessment

**Figure 5:**
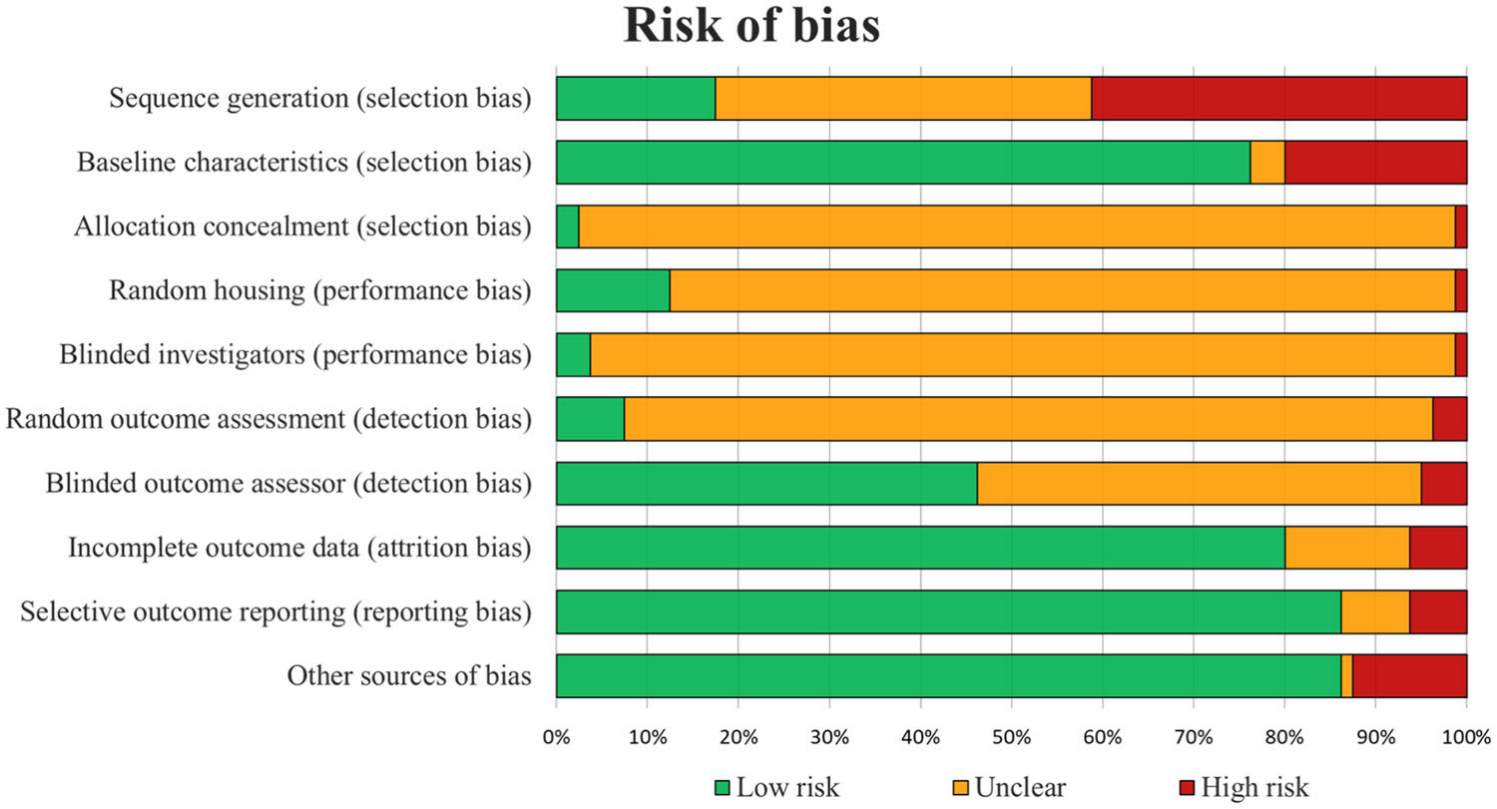
SYRCLE's risk of bias tool assessment of the included studies. The bar chart shows the percentage of all studies that met each quality item, scored as “Low risk”, “High risk”, or “Unclear”.

The SYRCLE's risk of bias tool assessment of the included studies found an 84% agreement between the two authors involved in the evaluation. 42% of items were rated as low risk of bias, 9% of items were rated as high risk of bias, and 49% of items were rated as unclear. The analysis of the risk of bias over time did not display a significant trend toward improving the quality of the included studies, with low‐risk items reported in 42% versu 41% in the most recent half of the papers vs. the older ones. The risk of bias assessment of all included studies is illustrated in Figure 5.

## DISCUSSION

The main finding of this systematic review of preclinical studies is that there is an increasing interest in the use of cell‐based injectable therapies for OA treatment alternative to the most common bone marrow and adipose tissue sources, with stronger evidence on cell therapies obtained from the umbilical cord and placenta/amniotic tissue. While expanded umbilical cord, MSCs reported promising disease‐modifying effects in preventing OA progression in animal models, placenta/amniotic tissue also reported negative effects on OA joints. Lower evidence has been found for other cellular sources such as embryonic, synovial, peripheral blood, dental‐pulp, cartilage, meniscus, and muscle‐derived products.

Over the past decade, growing interest in the clinical use of MSCs has led to exploring various sources. While bone marrow and adipose tissue are the most common due to their ease of extraction and beneficial properties, other MSC sources like umbilical cord blood, dental pulp, amniotic fluid, and placenta‐derived MSCs have also shown promise. These alternatives offer advantages like higher proliferative capacity, excellent regenerative potential, low immunogenicity, and non‐invasive collection [30, 43, 47, 52, 57]. However, comparative studies comparing different sources of MSCs showed contradictory results [1, 35, 36, 62]. Moreover, current evidence is limited to in vitro studies, which do not necessarily directly translate into effects at the joint level. This systematic review evaluated the disease‐modifying effects of these treatments in animal studies to provide objective evidence and support their clinical use.

Different sources of MSCs have been explored, but a synthesis of the disease‐modifying effects could only be carried out for umbilical cord, placenta, and synovial fluid‐derived MSCs. Umbilical cord‐derived injectable products have been the most investigated in animal studies, showing a 92.6% rate of positive disease‐modifying effects, with 83.4% of the studies reporting benefits at the cartilage level. These benefits have been observed through macroscopic, histological, and immunohistochemical analysis, reported in 71.4%, 82.6%, and 100% of cases, respectively. Similarly, expanded synovial fluid/membrane cells showed positive results, with 88.9% of the studies reporting a reduction of OA progression at macroscopic, histological, and immunohistochemical levels. On the other hand, placenta‐derived products have demonstrated some conflicting results. On one side, there were positive disease‐modifying effects, with 80% of the studies reporting benefits at the cartilage level. These benefits have been observed through macroscopic, histological, and immunohistochemical analysis, showing positive disease‐modifying effects in 75%, 78.6%, and 66% of the cases, respectively. On the other side, placenta‐derived products also led to some negative effects on OA joints, with an increase in synovitis and fibrosis after ASA injections, as documented in two studies [41, 42]. Looking at these results, greater attention must be paid to the clinical translation for placental‐derived products. Unfortunately, despite some promising results, evidence on the other cell sources is still scarce. Thus, further investigation into their clinical potential is warranted.

Imaging analysis, from simple radiography to MRI, showed more consistent results with UC‐MSCs, with seven out of eight studies reporting a positive effect compared to OA controls (micro‐CT in five studies, plain radiographs in three studies, and MRI in two studies). For placenta‐derived products, results were less consistent, with two out of four studies (Micro‐CT) reporting positive effects, one study reporting no significant changes, and one study reporting a worsening. Concerning the findings derived from histological and immunohistochemical analyses, these evaluations should be considered with more caution due to the limits of the imaging analyses in detecting cartilage and subchondral bone level changes, as previously documented in the previous ESSKA‐ORBIT studies [4, 18] as well as in clinical practice, where there is poor correlation between clinical outcome and imaging findings [79].

The comparison of the results of the current study with those obtained by the previous ESSKA‐ORBIT systematic reviews on adipose and bone‐marrow‐derived products showed that umbilical‐cord‐derived products reported similar disease‐modifying effects compared to adipose‐derived products and better than bone‐marrow products, although supported by a lower number of studies. In contrast, placenta‐derived products yielded inferior disease‐modifying effects to these treatments. More precisely, overall disease‐modifying effects were found in 94% of studies on adipose‐derived products, 93% for umbilical cord products, 85% for bone marrow, and 81% for placenta‐derived products. Interestingly, umbilical cord and placenta‐derived products showed better clinical performance, with 86% and 100% of papers reporting a significant improvement in treated animals versus 78% in both adipose and bone marrow groups, respectively. This may be due to the limited number of studies. Also, it may be speculated to be related to a better overall inflammatory response, reflected by an improved biomarker secretion profile reported in 100% and 90% of the placenta and umbilical cord‐derived products, respectively, versus 86% and 50% in adipose and bone‐marrow‐derived products, respectively. Finally, while over 80% of the included studies found improvement at the cartilage level, only 67% and 50% found the same effects on the synovium tissue in the umbilical cord and placenta‐derived products, respectively. These results are comparable to those reported in adipose‐derived products (60%) and superior to bone‐marrow products (30%).

Umbilical cord and placenta‐derived products have been a focus of interest for researchers, clinicians, and patients for years. These cell‐based products have a lower occurrence of replicative senescence, a higher proliferation rate than other MSC sources, robust immunomodulatory qualities, and are relatively easy to produce and readily available [29, 72, 74]. They also provide a solution for those who cannot have autologous tissue harvested due to contraindications or those who wish to avoid the harvesting procedure. Therefore, they seem ideal candidate sources for treating OA. This systematic review confirms that regenerative properties seen in in vitro studies in umbilical cord and placenta‐derived MSCs were translated into improved structural and clinical outcomes in animal models. Most of the immunomodulatory response was seen at the cartilage level and indirectly at the serum and synovial fluid levels. However, the current study's findings and previous studies in this series showed that MSCs from different sources had less effect on synovium [8, 56]. It has been demonstrated that MSCs show immunomodulatory activity in co‐culture studies, reducing inflammation and apoptosis. Still, this effect was not as pronounced in animal studies included in the previous ESSKA‐ORBIT reviews and the current study [8, 56, 82]. The dosage and timing of MSC injections could be key factors influencing the outcomes. The quantity of cells administered may have been optimal for cartilage while insufficient for producing a significant impact on the synovium, which suffers more from inflammatory infiltration and thus is more difficult to influence by treatments that rely on mechanisms of action directed against inflammation. It has been hypothesised that synovitis, especially at the early stages of OA, mediates OA structural and clinical OA progression [2, 54, 64]. However, studies that examined direct inhibition of inflammatory mediators such as IL‐1β, IL‐6, and TNFα found that they did not improve pain, synovitis, or OA progressions as assessed by MRI or ultrasound [16, 67, 69]. This ESSKA‐ORBIT review suggests that most clinical and structural positive effects could result from cartilage anabolic and anti‐inflammatory effects rather than a product of synovial tissue response. Regardless, only a handful of papers have examined the synovial effect of intraarticular MSC injection. R. Ferracini et al. investigated preclinical conditions in patients that may affect clinical outcomes following MSC treatment. They found that patients with predominant synovitis are prone to persistent pain 1‐year post‐treatment [25]. These results underscore the complexity of OA pathomechanism and potential therapies. Further research should be conducted on the role of synovium on OA progression in the different stages of the disease and its role in the response to cell‐based therapies.

Most of our knowledge of umbilical cord and placenta‐derived products stems from preclinical in vitro and animal studies. Results as objective as those reported in animals are difficult to reproduce in humans due to various ethical and practical concerns. In contrast, clinical outcomes are more difficult to assess in animal studies. Dhillon et al. [19] conducted a systematic review examining the clinical efficacy of umbilical cord‐derived products in treating OA. Overall, seven studies were included. They found umbilical cord‐derived products yielded clinical improvement in patients, but the literature lacks high‐quality randomised studies [19]. A recent systematic review by Di Matteo et al. [20] included 16 clinical studies on placenta‐derived products. The authors concluded that these products show a good safety profile and satisfactory results in treating OA. Another potential clinical use may be in the augmentation of surgical procedures. In patients with OA with axial deformity, a high tibial osteotomy (HTO) may be indicated. HRB Abd Razak et al. conducted a systematic review examining the efficacy of adipose and umbilical cord‐derived MSC therapy for early OA combined with HTO. They reviewed nine articles and reported good short‐term clinical results [60]. Similarly, Reale et al. [61] examined a broader spectrum of cartilage surgical treatments combined with cellular injective treatments. They found that injective treatments enhance pain relief and functional recovery in these patients [61]. However, the aforementioned clinical studies are heterogeneous in their preparation process and need more high‐quality evidence [20].

The current systematic review has several limitations. Firstly, the included papers exhibit heterogeneity across various aspects, including diverse methodologies employed for OA induction, differences in follow‐up periods and measurement criteria, various injection formulations and protocols, and the use of different animal models. The heterogeneous nature of the studies included may be explained by the relative novelty of MSC application in OA and the lack of clear treatment protocols and recommendations. Methodological heterogeneity can be improved by adherence to specific guidelines, that is, SYRCLE's tool. Addressing methodological heterogeneity alone can enhance the quality and reliability of results. Secondly, the prevalent utilisation of xenograft products, particularly those derived from umbilical and placental sources, raises concerns regarding potential immune responses and rejection in the treated animals, compromising effectiveness. Unlike allo‐ and auto‐grafts, the regenerative potential of xenografts is unclear, especially in humans. Furthermore, MSCs are commonly sourced from autografts and allografts in the clinical setting. Consequently, the translatability of the observed results to the clinical domain remains uncertain. Thirdly, in most cases, animal models did not follow the pathophysiology of OA development in humans, as it was mostly induced using chemical or mechanical methods. However, while different in physiology and composition, animal models allow us to examine the modulatory effects of MSCs on impaired cartilage in ways that are challenging in human trials (i.e., histological examination). It is essential, as MR imaging and radiographic evaluations, the most commonly used imaging evaluation tools in human trials, proved less reliable in assessing the effect of MSC treatment on cartilage tissue. In this light, preclinical studies remain important to understand how MSCs target and affect joint tissues affected by OA processes, giving useful indications for translating these cell‐based approaches into clinical practice [28]. Lastly, in their scoping review, Liu et al. [46] emphasised the lack of standardisation in MSC preparation devices and cellular characterisation, highlighting the need for improved reporting and standardised protocols in future studies.

## CONCLUSIONS

There is an increasing interest in using cell‐based injectable therapies for OA treatment alternative to the most common bone marrow and adipose tissue sources, with higher evidence on cell therapies obtained from umbilical cord and placenta/amniotic tissue. While expanded umbilical cord, MSCs reported promising disease‐modifying effects in preventing OA progression in animal models, placenta/amniotic tissue also reported deleterious effects on OA joints. Lower evidence has been found for other cellular sources such as embryonic, synovial, peripheral blood, dental pulp, cartilage, meniscus, and muscle‐derived products.

## AUTHOR CONTRIBUTIONS

**Yosef Sourugeon**: Writing; investigation. **Angelo Boffa**: Investigation; formal analysis; resources. **Carlotta Perucca Orfei**: Investigation. **Laura de Girolamo**: Conceptualization; review & editing; methodology. **Jeremy Magalon**: Review & editing. **Mikel Sánchez**: Review & editing. **Thomas Tischer**: Conceptualization; review & editing. **Giuseppe Filardo**: Project administration. **Lior Laver**: Supervision.

## CONFLICT OF INTEREST STATEMENT

The authors declare no conflicts of interest.

## ETHICS STATEMENT

This material is the authors' own original work, which has not been previously published elsewhere. The paper is not currently being considered for publication elsewhere. The paper reflects the authors' own research and analysis in a truthful and complete manner. The paper properly credits the meaningful contributions of co‐authors and co‐researchers. The results are appropriately placed in the context of prior and existing research. All sources used are properly disclosed (correct citation). Literally copying of text must be indicated as such by using quotation marks and giving proper reference. All authors have been personally and actively involved in substantial work leading to the paper, and will take public responsibility for its content.

## Supporting information

Supporting information.

## Data Availability

Data supporting this study are included within Supporting Information (Table 1).
