# Supplementary material for: Cell‐based therapies have disease‐modifying effects on osteoarthritis in animal models: A systematic review by the ESSKA Orthobiologic Initiative. Part 3: Umbilical cord, placenta, and other sources for cell‐based injectable therapies
Source: Knee Surg Sports Traumatol Arthrosc. 2024 Sep 20;33(5):1695–708. doi: 10.1002/ksa.12472 (PMC12022835; doi:10.1002/ksa.12472)
Supplement: Supplementary file 1 — Supporting information. [file KSA-33-1695-s001.docx]

**Supplementary table 1.** Characteristics of the included studies on cell-based injectable products (other than bone marrow and adipose-derived).

| **Authors Journal Year** | **Animal Model** | **Evaluated  Joint** | **OA Model** | **Treatment groups** | **MSCs source  and origin** | **Expanded or Point of care** | **MSCs dose** | **Additional  procedure** | **Injection Protocol** N. injections Injection timing Injected Volume | **Follow-up** | **Final Results** |
| --- | --- | --- | --- | --- | --- | --- | --- | --- | --- | --- | --- |
| Bhattacharjeea et al. Proc Natl Acad Sci 2022 [6] | 24 Rats | Knee | Collagenase  injection | MSC (n=6) MSC + hydrogel (n=6) MSC + ASC (n=6 OA control (n=6) | Amniotic membrane  Allogeneic (Rat) | Point of care | - | Hydrogel for Amnion | Single injection - 100 μL | 1 month | Both amniotic membrane and adipose tissue groups showed comparable disease-modifying effects. Combined therapy showed synergistic effects. |
| Veronesi F et al. Int Orthop 2021 [88] | 12 Sheep | Knee | Bilateral  Meniscectomy | MSC (n=6 knees) ASC (n=6 knees) SVF (n=6 knees) OA control (n=6 knees) | Amnion  Xenogeneic (Human) | Expanded | 2.5 x 10^6^ | - | Single injection - 1 ml | 3 months | All treatments provided better biochemical, gross, and biomechanical results compared to the control group. |
| Kimmerling KA et al. J Orthop Re 2020 [49] | 40 Rats | Knee | MIA injection | High dose MSC (n=10) 50 μL Low dose MSC (n=10) 25 μL Steroid (n=10) OA control (n=10) | Amnion  Xenogeneic (Human) | Point of care | - | - | Single injection - High dose: 50 μL Low dose MSCs: 25 μL | 2 weeks | High dose MSC treatment resulted in clinical improvement of rats suffering from MIA induced OA. |
| Reece DS et al. Tissue Eng Part A 2020 [72] | 36 Rats | Knee | Medial Meniscus destabilization | µ‐dHACM (n=9) RPS µ‐dHACM (n=9) OA control (n=9) Sham control (n=9) | Amniotic membrane  Xenogeneic (Human) | Point of care | 80mg/mL | - | Single injection - 50 µL | 3 weeks | Reduced Size Profile of Amniotic Membrane Particles Decreases Osteoarthritis Therapeutic Efficacy. |
| Zavatti M et al. Biofactors 2020 [104] | 20 Rats | Knee | MIA injection | MSC (n=8) Exosomes (n=8) OA control (n=4) | Amniotic fluid  Xenogeneic (Human) | Expanded | 5 x 10^5^ | - | Single injection - 50 μL | 3 weeks | MSCs and exosomes have shown similar effect, alleviating histological OA progression. |
| Marino-Martinez IA et al. Exp Ther Med 2019 [56] | 6 Rabbits | Knee | Collagenase  injection | MSC (n=6 knees) OA control (n= 6 knees) | Amniotic membrane  Xenogeneic (Human) | Point of care | 0.040 mg/0.200 ml | Amniotic membrane  lyophilized and pulverized | Single injection - 0.2 ml | 6 weeks | Intra‑articular injection of human amniotic membrane delayed histological changes of cartilage in OA. |
| Salazar-Noratto GE et al. Regen Eng Transl Med 2019 [75] | Rats | Knee | Medial Meniscus destabilization | MCS OA control | Amniotic membrane  Xenogeneic (Human) | Point of care | 40mg/ml | - | Single injection - 50 µL | 3 weeks | Placental MSCs had immunomodulatory effect on the synovium. |
| Raines AL et al. Tissue Eng Part A 2017 [70] | 54 Rats | Knee | Medial Meniscus destabilization | High dose MSC (n=18) Low dose MSC (n=18) OA control (n=18) | Amniotic membrane  Xenogeneic (Human) | Point of care | 50/100 µg/µL | - | Single injection - 50/100 µg/µL | 1 month | MSCs tissue may attenuate progressive cartilage degeneration in a small animal model of OA with a dose dependent effect. |
| Tanrisever M et al. Turkish J Vet Anim Sci 2017 [85] | 21 Rabbits | Knee | ACL transection | MSC (n=7) HA (n=7) OA control (n=7) | Amniotic fluid  Xenogeneic (Cox) | Point of care | - | - | Three injections 1 -week interval 0.5 ml | 3 months | MSC treatment alleviated microscopic and macroscopic progression of OA. |
| Willett NJ et al. Arthritis Res Ther 2014 [96] | 10 Rats | Knee | Medial meniscal transection | MSC (n=5) OA control (n=5) | Amniotic membrane  Xenogeneic (Human) | Point of care | - | - | -   -   - | 3 weeks | Injections of a devitalized allograft derived from amniotic membrane induced a reduction in cartilage degeneration. |
| Desai S et al. Front Bioeng Biotech 2022 [22] | Rats | Knee | Meniscal injury | MSC BMMSC OA control | Cartilage  Xenogeneic (Human) | Expanded | 1.6 x 10^6^ | - | Two injections 3-week interval 50µl | 7 weeks | Both treatment groups ameliorated OA progression, with cartilage progenitor cells being significantly better than BMMSCs. |
| Fan MP et al. Eur Rev Med Pharmacol Sci 2018 [29] | 30 Rat | Knee | Partial medial meniscectomy | MSC (n=10) OA control (n=10) Healthy control (n=10) | Cartilage  Allogeneic (Rat) | Expanded | 5 x 10^6^ | - | Single injection - 100 µl | 2 months | MSC treatment downregulated inflammatory response. |
| Xia QQ et al. Stem Cells Transl Med 2015 [98] | 44 Mice | Knee | ACL transection | MSC-Asttrin (n=11) MSC-GFP (n=11) OA control (n=11) Healthy control (n=11) | Cartilage  Xenogeneic (Human) | Expanded | 1 x 10^5^ | Atsttrin/GFP  transduction | Single injection - 10 μL | 2 months | Transduced MSCs alleviated histological OA progression, with Atsttrin transduced cells achieving significant results as early as 4 weeks. |
| Correa Maldonado D et al. Eur Rev Med Pharmacol Sci 2021 [19] | 36 Rats | Knee | Zymosan  injection | MSC (n=6) Saline gavage (n=6) Glucosamine-chondroitin gavage (n=6) Diacerein gavage (n=6) OA control (n=6) Healthy control (n=6) | Dental Pulp  Xenogeneic (Human) | Expanded | 8 x 10^5^ | N/A | 1 injection - 4 ml | 2 months | Intra-articular dental pulp MSCs provides significant improvement in tissue regeneration in an OA model. |
| Xing D et al. Molecules 2021 [100] | 28 Rats | Knee | ACL transection | Multiple injections MSC (n=10) Single injection MSC (n=10) OA control (n=8) | Embryoid bodies  Xenogeneic (Human) | Expanded | 1 x 10^6^ | - | Single or three injections 1-week interval 100 μL | 10 weeks | MSC injection reduced macro and microscopic OA progression. Multiple injection protocol yielded better overall results. |
| Jiang B et al. Theranostics 2019 [39] | 8 Monkeys | Knee | Natural occuring | MSC spheroids (n=3) BMMSC (n=3) OA control (n=2) | Embryonic  Xenogeneic (Human) | Expanded | 1 x 10^7^ cells | Spheroid formation | Three injections - 100 µL | 9 months | Embryonic MSCs and BMMSCs improved clinical performance of spontaneous OA in rhesus macaques. |
| Chang YH et al. Stem Cells Int 2020 [14] | 5 Rabbits | Knee | ACL transection | MSC (n=3 knees) OA control (n=3 knees) Healthy control (n=4 knees) | Epidermal  keratinocytes  Xenogeneic (Human) | Expanded | 1.8 x 10^8^ | - | Single injection 0.5 ml | 3 months | MSCs decreased catabolic markers and slowed histologic OA progression. |
| Shen WL et al. Stem Cells Transl Med 2014 [81] | 6 Rats | Knee | Partial medial meniscectomy | MSC (n=6 knees) OA control (n=6 knees) | Meniscus  Xenogeneic (Human) | Expanded | 6 x 10^6^ | - | Single injection - 50 μL | 3 months | MSCs derived from meniscal progenitor cells ameliorated OA progression and induced early meniscal regeneration. |
| Shen WL et al. Stem Cells Dev 2013 [82] | 9 Rabbits | Knee | Partial medial meniscectomy | MSC (n=9 knees) OA control (n=9 knees) | Meniscus  Allogeneic (Rabbit) | Expanded | 6 x 10^6^ | - | Single injection - 100 μL | 3 months | MSCs derived from meniscal progenitor cells ameliorated OA progression, induced early meniscal regeneration and improved mechanical properties of cartilage. |
| Chan DC et al. J Orthop Res 2017 [13] | 21 Mice | Knee | Streptozotocin induced | MSC (n=7) Placebo (n=7) OA control (n=7) | Muscle  Xenogeneic (Human) | Expanded | 5 x 10^5^ | - | 4 injections 1-week interval 10 µl | / | MSC transplantation effectively ameliorated cartilage degeneration in a diabetes-related OA mouse model. |
| Mifune Y et al. Osteoarthritis Cartilage 2013 [58] | 36 Rats | Knee | MIA injection | MSCs (n=6) MSC + PRP (n=6) sFlt-1-MSC + BMP4-MSC (n=6) sFlt-1-MSC + BMP4-MSC + PRP (n=6) PRP (n=6) OA control (n=6) | Muscle  Allogeneic (Mouse) | Expanded | 5 x 10^5^ | Retroviral transduction of BMP4 and Flt1 | Single injection - 50 μL | 3 months | MSC + PRP injection alleviated OA progression, PRP further promoted therapeutic effects of MSC groups at 4-week time. |
| Matsumoto T et al. Arthritis Rheum 2009 [57] | 60 Rats | Knee | MIA injection | MSC (n=12) sFlt-1-MSC + BMP4-MSC (n=12) VEGF-MSC + BMP4-MSC (n=12) BMP4-MSC (n=12) OA control (n=12) | Muscle  Allogeneic (Mouse) | Expanded | 5 x 10^5^ | Retroviral transduction of BMP4, Flt1 and VEGF | Single injection - - | 4 months | MSC-based therapy involving sFlt-1 and BMP-4 repairs are more effective the MSC-based therapy for OA treatment. |
| Zhang LS et al. Cell and Bioscience 2021 [107] | Rabbits | Knee | MIA injection | MSC MSC + HA HA OA control Sham control | Not specified  Xenogeneic (Human) | Expanded | 2 x 10^6^ | Hydrogel | Three injections 1-week interval 200 μL | 2 months | Combined Hydrogel and MSC treatment alleviated macro and microscopic OA progression. |
| Zhu YX et al. Bmc Biotechnology 2016 [110] | 13 Rats | Knee | MIA injection | MSC (n=5) Chondrogenic MSC (n=5) OA control (n=3) | Not specified  Xenogeneic (Human) | Expanded | 1 × 10^6^ | Chondrogenic induction | Single injection - 500 μL | 4 months | Promising results obtained from cell transplantation using MSCs. The effects are more evident when cells are pre-differentiated towards the chondrogenic lineage. |
| Lin W et al. Biochem Biophys Res Commun 2022 [52] | 17 Mice | Knee | Meniscal  destabilization | MSC (n=4) ASC (n=4) OA Control (n=4) Sham control (n=5) | Peripheral  Blood  Allogeneic (Mouse) | Expanded | 5 x 10^5^ | - | Three injections 1-week interval 10 µl | 2 months | Injections of peripheral blood MSCs and ASCs alleviated OA progression. |
| Broeckx SY et al. Equine Vet J 2019 [11] | 12 Horses | Metacarpo- phalangeal  joint | Osteochondral  chip fragment | Chondrogenic MSC (n=6) OA control (n=6) | Peripheral  Blood  Allogeneic (Horse) | Expanded | 2 x 10^6^ | Chondrogenic induction | Single injection - 2 ml | 3 months | Equine allogeneic chondrogenic-induced MSCs combined with equine allogeneic plasma may be a promising treatment for OA in horses. |
| Daems R et al. Stem Cells Int 2019 [20] | 6 Dogs | Elbow | Natural occurring | MSC (n=6) Placebo (n=6) | Peripheral  Blood  Xenogeneic (Horse) | Expanded | 1 x 10^6^ | Chondrogenic induction | 1 injection - 0.5 ml | 3 months | MSC injection reduces pain and lameness according to owner surveys. |
| Deng MW et al. Cell Transplant 2015 [21] | 28 Rats | Knee | Papain/cysteine  injection | MSC + HA (n=10) gm-MSC + HA (n=18) HA (n= 10) | Peripheral  Blood  Allogeneic (Rat) | Expanded | 5 x 10^6^ | - | 1 injection - 100 µl | 6 weeks | MSC + HA injection inhibit progression of OA when compared with HA monotherapy. |
| Fan M et al. Front Pharmacol 2022 [28] | 62 Rats | Knee | ACL transection | MSC (n=18) MSC-CM (n=8) OA control (n=18) Healthy control (n=18) | Placenta  Xenogeneic (Human) | Expanded | 1.6 x 10^6^ | - | Four injections 1-week interval 50µl | Unclear (2 months?) | MSCs were effective in slowing OA progression |
| Kimmerling KA et al. Arthritis Res Ther 2022 [49] | 100 Rats | Knee | Meniscal injury | high-dose ASA (n=25) low-dose ASA (n=25) FGF18 (n=25) OA control (n=25) | Placenta  Xenogeneic (Human) | Point of care | - | - | Single injection - high dose: 50 µl low dose: 25 µl | 3 weeks | ASA exhibited short term improvements in cartilage properties that were lost in the long term. However, pain and function were improved. |
| Kwapisz A et al. Am J Sports Med 2022 [50] | 24 Guinea Pigs | Knee | Natural Occurring | MSCs + HA (n=8) ASCs + HA (n=8) HA (n=8) Control (n=8) | Placenta  Xenogeneic (Human) | Expanded | 5 x 10^5^ | - | Three injections Two-month interval 100 μL | 6 months | Intra-articular injection of MSCs and HA had similar clinical effect on mitigating OA progression. |
| Veronesi F et al. Biology 2022 [88] | 24 Sheep | Knee | Meniscectomy | MSC (n=6) ASCs (n=6) SVF (n=6) OA control (n=6) | Placenta  Allogeneic (Sheep) | Expanded | 2.5 x 10^6^ | - | Single injection - 1ml | 6 months | All the three treatments showed better results than control (injection of NaCl), but SVF and placental MSCs showed superiority over ASCs. |
| Flannery CR et al. Osteoarthritis Cartilage 2021 [30] | 84 Rats | Knee | Medial Meniscus destabilization | Single inj. - MSC High dose (n=12) 50 mg/ml Single inj. - MSC Low dose (n=12) 25 mg/ml Two inj. - MSC High dose (n=12) 50 mg/ml Two inj. - MSC Low dose (n=12) 25 mg/ml Single inj. OA steroids (n=12) Two inj. OA saline (n=12) Single inj. OA saline (n=12) | Placental tissues  Xenogeneic (Human) | Point of care | (50mg/ml), or  (25 mg/ml) of  PTP-001 | - | Single or two injections - 50 µl | 6 weeks | MSC treatment improved clinical presentation in rat OA model. High dose multi-injection protocol improved histopathological scores. |
| Sampath SJP et al. Stem Cell Investig 2021 [76] | 30 Rats | Knee | MIA injection | MSC (n=6)  MSC + stigmasterol (n=6) Stigmasterol (n=6) OA control (n=6) Healthy control (n=6) | Placenta  Xenogeneic (Human) | Expanded | 2 x 10^6^ | - | Single injection - - | 1 month | MSCs + stigmasterol treatment achieved significant histological alleviation of OA progression. |
| Wang AT et al. Front Bioeng Biotechnol 2020 [93] | 30 Rats | Knee | MIA injection | MSC (n=6) MSC + HA (n=6) HA (n=6) OA control (n=6) Healthy control (n=6) | Placenta  Xenogeneic (Human) | Expanded | 1 x 10^6^ | - | Two injections 4-week interval 100 μL | 2 months | MSC treatment alleviated histological OA progression and had anti-inflammatory effects. Best results were reached with HA+MSC treatment. |
| Kim JK et al. Biol Pharm Bull 2010 [44] | Rats | Knee | MIA injection | High dose HPE Medium-High dose HPE Medium dose HPE Low dose HPE OA control | Placenta  Xenogeneic (Human) | Point of care | 0.022, 0.066, 0.2 or 0.4 ml/kg | - | 14 injections 1-week interval - | 1 month | HPE significantly reduced deformity of knee joints and suppressed the histological change in MIA-induced OA. |
| Nabavizadeh SS et al. J Orthop Surg Res 2022 [60] | 36 Rats | Knee | Collagenase  injection | MSC (n=6) MSC + secrotome (n=6) MSC + PRP (n=6) MSC + PRP + secrotome (n=6) HA (n=6) OA control (n=6) | Synovial membrane  Allogeneic (Rat) | Expanded | 5 x 10^6^ | - | Single injection - 50 μL | 3 months | The combination of the synovial fluid MSCs/secretome and PRP had a considerable effect on articular cartilage preservation. |
| Alahdal M et al.  Front Immunol 2021 [1] | 40 Rats | Knee | Medial Meniscus destabilization | MSC + IDO1 (n=10) MSC + Epacadostat (n=10) OA control (n=10) Sham control (n=10) | Synovial fluid   Xenogeneic (Human) | Expanded | 1 x 10^6^ | - | Four injections 2-week interval 100 μL | 2 months | SF MSC are an effective tool to improve cartilage repair however IDO1 impairs chondrogenic potential of MSCs and slows down cartilage regeneration. |
| Estakhri F et al. Iran J Med Sci 2021 [27] | 48 Rats | Knee | ACL transection | MSC (n=6) MSC + apigenin 0.3µM (n=6) MSC + apigenin 0.1µM (n=6) apigenin 0.13M (n=6) apigenin 0.1µM (n=6) HA (n=6) OA control (n=6) Sham control (n=6) | Synovial membrane   Allogeneic (Rat) | Expanded | 3 x 10^6^ | - | Three injections 1-week interval 50µl | 3 months | MSCs had immunomodulatory effect on OA, with greater therapeutic effect when apigenin high dose was added. |
| Horiuchi K et al. Sci Rep 2021 [37] | 40 Rats | Knee | Medial Meniscus destabilization | Cultured MSC (n=12) Thawed MSC (n=12) OA control (n=24) | Synovial tissue  Allogeneic (Rat) | Expanded | 1 x 10^6^ | Deep freeze (Thawed) | Six injections 1 -week interval 50 µl | 2 months | Thawed cryopreserved MSCs and cultured MSCs showed a comparable inhibitory effect on OA progression in a rat OA model. |
| Kim YS et al. Knee 2021 [47] | 60 Dogs | Knee | ACL transection | High dose MSC (n=12) Medium dose MSC (n=12) Low dose MSC (n=12) OA control (n=12) Sham control (n=12) | Synovial tissue  Xenogeneic (Human) | Expanded | Low dose: 2.4 x 10^6^  Medium dose: 4.8 x 10^6^  High dose: 9.6 x 10^6^ | - | Single injection - - | 21 month | MSC injection ameliorated the histopathological progression of canine OA. No differences were found between different doses. |
| Qiong J et al. Eur Rev Med Pharmacol Sci 2020 [69] | 24 Rats | Knee | Partial medial meniscectomy | MSC (n=8) OA control (n=8) Healthy control (n=8) | Synovial tissue  Allogeneic (Rat) | Expanded | 5 x 10^6^ | - | Single injection - - | 2 months | MSCs therapy alleviated inflammation resulted from OA and protected cartilage from OA damage. |
| Zare R et al. Int J Rheumatol 2020 [103] | 28 Rats | Knee | Collagenase  injection | MSCs (n=7) ASCs (n=7) HA (n=7) OA control (n=7) | Synovial membrane  Allogeneic (Rat) | Expanded | 2.5 x 10^6^ | - | Single injection - 50 μL | 3 months | MSCs alleviated radiographic OA progression, showing a superior effect over HA. |
| Neybecker P et al. Stem Cell Res The 2018 [62] | 48 Rats | Knee | ACL transection | MSC (n=16) OA control (n=16) Sham control (n=16) | Synovial fluid   Xenogeneic (Human) | Expanded | 1 x 10^6^ | - | Two injections 1- week interval 50 μL | 2 months | Synovial fluid MSC injection did not produce any beneficial or detrimental effects. |
| Ozeki N Osteoarthritis Cartilage 2016 [63] | 76 Rats | Knee | ACL transection | Weekly MSC injections Single MSC injection Weekly saline injections | Synovial fluid   Xenogeneic (Human) | Expanded | 1 x 10^6^ | - | Single or 12 injections 1 -week interval 50 μL | 3 months | Periodic injections of synovial MSCs inhibited OA progression more than single injection of MSC. |
| Hatsushika D et al. Osteoarthritis Cartilage 2014 [34] | 10 Pigs | Knee | Medial Meniscus destabilization | MSC (n=5) OA control (n=5) | Synovial tissue  Allogeneic (Pig) | Expanded | 5 x 10^7^ | - | Three injections 1 -week interval 1 ml | 4 months | Allogeneic synovial MSCs appeared to promote meniscus regeneration and provide articular cartilage protection. |
| Bie Y et al. Immunoph Immunotox 2023 [7] | 30 Rabbits | Knee | Collagenase  injection | MSC (n=6) MSC + Rapamycin (n=6) Rapamycin (n=6) OA control (n=6) Healthy control (n=6) | Umbilical cord  Xenogeneic (Human) | Expanded | 1 x 10^7^ | - | Single injection - 0.5 ml | 6 weeks | All treatments alleviated OA progression. Rapamycin + MSCs showed synergistic effect. |
| Chang YH et al. Biomedicines 2022 [15] | 9 Mice | Knee | Collagenase  injection | MSC + exosome depleted FBS (n=3) MSC + FBS (n=3) Saline (n=3) | Umbilical cord  Xenogeneic (Human) | Expanded | 4 x 10^5^ | - | Single injection - 50µl | 1 month | Exosome depleted FBS + MSCs alleviated microscopic OA progression and had overall better results vs MSCs cultured in FBS. |
| Chen P et al. International Journal of Pharmaceutics 2022 [16] | 24 Rats | Knee | ACL transection | MSC (n=6) MSC-exosomes (n=6) OA control (n=6) Sham control (n=6) | Umbilical cord  Xenogeneic (Human) | Expanded | 5 x 10^5^ | - | Four injections 1-week interval 200 μL | 2 months | Both treatment groups ameliorated macro and microscopic OA progression. Both had similar clinical effect. |
| Ju Y et al. Acta Histochem 2022 [41] | 52 Rats | Knee | ACL transection | single dose MSC (n=9) repeated MSC (n=8) single dose ASC (n=9) repeated dose ASC (n=8) OA control (n=9) Sham control (n=9) | Umbilical cord  Xenogeneic (Human) | Expanded | 1.6 x 10^6^ | - | Single or two injections 2-week interval 50µl | 3 months | All treatment groups ameliorated OA progression. No difference between cell source or number of injections. |
| Kim M et al. Tissue Eng Regen Med 2022 [45] | 27 Goats | Knee | ACL transection + medial menscectomy | MSC (n=6) MSC + CAM (n=6) CAM (n=6) OA control (n=6) Healthy control (n=3) | Umbilical cord  Xenogeneic (Human) | Expanded | - | - | - | 12 months | MSCs improved clinical and radiographic results at 6-month time post injection. Synergism with CAM was recorded only on gross morphology. |
| Liu J et al. Front Endocrinol 2022 [53] | 24 Rabbits | Knee | Papain injection | MSC (n=6) MSC + GO (n=6) GO (n=6) Control (n=6) | Umbilical cord  Xenogeneic (Human) | Expanded | 5 x 10^6^ | - | Single injection - 10ml | 2 months | MSCs have shown a reduction in joint and systemic inflammation and had a synergistic effect with GO granules. |
| Wang AT et al. Bioact Mater 2022 [94] | 40 Rats | Knee | ACL transection | High dose MSC (n=8) Low dose MSC + microniches (n=8) HA (n=8) OA Control (n=8) Healthy control (n=8) | Umbilical cord  Xenogeneic (Human) | Expanded | Low dose: 1 x 10^5^ High dose: 1 x 10^6^ | Microniche - 3D gelatin microcarriers | Single or four injections 1-week interval 200 μL | 3 months | Single low dose MSC injection with microniches had similar effect on OA as high dose, multiple injections of MSCs. Both alleviated microscopic and radiographic OA progression. |
| Bertoni L et al. Int J MoL Sci 2021 [5] | 8 Horses | Fetlock  joint | Osteochondral  chip fragment | Group 1: MSC (n=5) BMMSC (n=5) OA control (n=5) Group 2: BMMSC (n=3) OA control (n=3) | Umbilical cord  Allogeneic (Horse) | Expanded | 10 x 10^6^ | - | Single injection 2 ml | 3 months | No significant differences in the development of OA pathological changes were observed between umbilical MSC group and other groups. |
| Chang YH et al. Stem Cells Int 2021[15] | 6 Rabbits | Knee | ACL transection | MSC + HA (n=6) OA control (n=6) | Umbilical cord  Xenogeneic (Human) | Expanded | 1 x 10^7^ | - | Single injection - 500 μL | 3 months | HA+MSC treatment slowed early MRI OA progression and slowed microscopic OA progression. |
| Mostafa A et al.  J Microsc Ultrastruct 2021 [59] | 30 Rats | Knee | MIA injection | IA MSCs (n=6) IV MSCs (n=6) OA control (n=6) OA + IA saline (n=6) Non OA + IV saline (n=6) | Umbilical cord  Xenogeneic (Human) | Expanded | 1 x 10^6^ | - | Single injection - 50 μL | 1 month | IA injection of MSCs slowed histopathological progression of OA. |
| Tang S et al.  Int J Nanomedicine 2021 [84] | 24 Rats | Knee | ACL transection | MSCs (n=6) MSC-exosomes (n=6) OA control (n=6) Healthy control (n=6) | Umbilical cord  Xenogeneic (Human) | Expanded | 5 x 10^5^ | - | Single injection - 200 μL | 2 months | MSC and their exosomes exhibited similar effects, inhibiting OA progression via immunomodulation. |
| Tang S et al. Int J Nanomedicine 2021 [84] | 24 Rats | Knee | ACL transection | MSCs (n=6) MSCs-exosomes (n=6) OA control (n=6) Normal (n=6) | Umbilical cord  Xenogeneic (Human) | Expanded | 5 x 10^5^ | - | Single injection - 200 μL | 3 months | MSCs and their derivatives ameliorated OA progression through reduction of inflammation. |
| Wang AT et al. Stem Cells Int 2021 [92] | 28 Rabbits | Knee | MIA injection | High dose MSC + Hydrogel-HA (n=4) Mid dose MSC + Hydrogel-HA (n=4) Low dose MSC + Hydrogel-HA (n=4) Hydrogel-HA (n=4) HA (n=4) OA control (n=4) Healthy control (n=4) | Umbilical cord  Xenogeneic (Human) | Expanded | High dose MSC: 0.1 x 10^7^ Medium dose MSC: 0.5 x 10^7^ Low dose MSC: 1 x 10^7^ | Hydrogel | Single injection - 100 μL | 3 months | MSC + HA/PG achieved histologic OA progression alleviation, immunomodulatory effect, and had overall better results vs HA or HA/PG treatment. Best results were with the mid dose group. |
| Wang XD et al. Int Orth 2021[95] | 24 Rabbits | Knee | Papain/cysteine  injection | MSC (n=6) MSC + GO granules (n=6) GO granules (n=6) OA control (n=6) | Umbilical cord  Xenogeneic (Human) | Expanded | 5 x 10^6^ | - | Single injection - 0.5 ml | 2 months | MSC treatment had immunomodulatory effect on OA model, MSC+GO therapy yielded better results vs MSC monotherapy. |
| Wu JL et al. Appl Sci 2021 [97] | 27 Rats | Knee | MIA injection | MSC (n=7) MSC + HA (n=7) HA (n=6) OA control (n=7) | Umbilical cord  Xenogeneic (Human) | Expanded | 2.5 x 10^6^ | - | Single injection - 100 μL | 4 months | MSC injection alleviated histological OA progression, MSC+HA had greater therapeutic effect. |
| Zhang Q et al. Bone & Joint Research 2021 [108] | 28 Rats | Knee | MIA injection | MSC (n=7) HA (n=7) OA control (n=7) Sham control (n=7) | Umbilical cord  Xenogeneic (Human) | Expanded | 2.5 x 10^5^ | - | Three injections 1-week interval 50 μL | 6 weeks | Multiple intra-articular administrations of MSCs exerted a significant therapeutic effect on severe OA. |
| Zhang XG et al. Chem Eng J 2021 [109] | 48 Mice | Knee | Medial Meniscus destabilization | High dose MSC (n=8) Low dose MSC (n=8) Low dose MSC+Microgel (n=8) Microgel (n=8) OA control (n=8) Healthy control (n=8) | Umbilical cord  Xenogeneic (Human) | Expanded | Low dose: 3 x 10^4^ High dose: 3 x 10^5^ | 3d microcryogel  impregnation | Single injection - 5 μL | 2 months | MSCs can be used to effectively overcome the OA progression and this effect can be enhanced by the use of a microgel. |
| Geng YY et al. J Bone Miner Metab 2020 [31] | 16 Rats | Knee | Medial Meniscus destabilization | mir-450 MSC (n=4) Zsgreen-MSC (n=4) OA control (n=4) Sham control (n=4) | Umbilical cord  Xenogeneic (Human) | Expanded | 1 x 10^7^ | Retroviral transduction of Zsgreen or miR-140 | Single injection - 100 µl | 2 months | mir-140-5p induced MSCs alleviated histological OA progression in OA model. |
| Lamers K et al. Vet Immunol Immunopathol 2020 [51] | 23 Sheep | Knee | Medial Meniscus destabilization | MSC (n=8) OA control (n=9) Sham + MSC (n=6) | Umbilical cord  Xenogeneic (Human) | Expanded | 1 x 10^7^ | - | Single injection - 1 ml | 3 months | The study indicates an immune response against the cell therapy, but this response was not accompanied by notable pathological changes. |
| Perry J et al. Osteoarthr Cartil Open 2020 [64] | 91 Mice | Knee | Partial medial  meniscectomy | Early injection MSC (n=22) Late injection MSC (n=25) OA control (n=27) Sham control (n=15) | Umbilical cord  Xenogeneic (Human) | Expanded | 5 x 10^5^ | - | Single injection - 10 μL | 3 months | Transplanted MSCs did not recover joint OA damage induced by the partial meniscectomy as assessed histologically. |
| Sofia V et al. Open Access Maced. J Med Sci 2020 [83] | 32 Rats | Knee | MIA injection | MSC (n=16) OA control (n=16) | Umbilical cord  Xenogeneic (Human) | Expanded | 1 x 10^6^ | - | Single injection - 50 μL | 3 weeks | MSC treatment significantly enhanced serum IL-10 levels. |
| Tong W et al. J Orthop Translat 2020 [86] | Rats | Knee | MIA injection | Multiple injection MSC Single injection MSC Multiple Chondrocyte injections OA control Healthy control | Umbilical cord  Xenogeneic (Human) | Expanded | 2.5 x 10^5^ | - | Single or three injections 1 week interval 50 μL | 1 month | MSC treatment alleviated OA progression. Multiple injections had better results vs single injection. |
| Xing D et al. Cell Transpl 2020 [99] | 32 Rats | Knee | ACL transection | High dose MSC (n=8) Low dose MSC (n=8) Low dose MSC+Microgel (n=8) OA control (n=8) | Umbilical cord  Xenogeneic (Human) | Expanded | Low dose: 1 x 10^5^ High dose: 1 x 10^6^ | Microcryogel discs | Single injection - 100 μL | 2 months | Injection of microgels loaded with a low dose of MSCs had similar therapeutic effects as a high dose of free MSCs, which decelerated the progression of OA. |
| Xing D et al. International Journal of Rheumatic Diseases 2020 [101] | 18 Rats | Knee | ACL transection | MSC+HA (n=6) HA (n=6) OA control (n=6) | Umbilical cord  Xenogeneic (Human) | Expanded | 1 x 10^6^ | - | Single injection - 100 μL | 3 months | A single injection of MSCs + HA can have temporary effects that decelerate the progression of cartilage destruction in a rat OA model. |
| Yan L et al. Cell Death & Disease 2020 [102] | 50 Rats | Knee | MIA injection | MSC (n=10) MSC + Platelet lysate (n=10) Platelet lysate (n=10) OA control (n=10) Healthy control (n=10) | Umbilical cord  Xenogeneic (Human) | Expanded | 1 x 10^6^ | - | Four injections 1-week interval 50 μL | 1 month | MSC treatment alleviated microscopic OA progression and improved clinical outcomes. |
| Endrinaldi E et al. Open Access Maced J Med Sci 2019 [25] | 32 Rats | Knee | MIA injection | MSC (n=16) Control (n=16) | Umbilical cord  Xenogeneic (Human) | Expanded | 1 x 10^6^ | - | Single injection - 50 µl | 3 weeks | MSCs significantly lowered iNOS serum levels in the rat OA model. |
| Kim H et al. Sci Rep 2019 [43] | 25 Rabbits | Temporo- mandibular  joint | MIA injection | MSCs - low dose (n=5) MSCs - medium dose (n=5) MSCs - high dose (n=5) Dexamethasome (n=5) OA control (n=5) Sham control (n=5) | Umbilical cord  Xenogeneic (Human) | Expanded | Low dose: 1 x 10^5^ Medium dose: 5 x 10^5^  High dose: 1 x 10^6^ | - | Single injection - 200 μL | 2 months | MSCs may be an effective treatment option for the treatment of OA. |
| Kim SE et al. Front Vet Sci 2019 [46] | 55 Dogs | Elbow | Natural occuring | MSC (n=30) OA control (n=25) | Umbilical cord  Allogeneic (Dog) | Expanded | 7 x 10^6^ | - | Single injection - 0.5 ml | 6 months | This study demonstrated improvement of clinical signs related to elbow OA in dogs treated with intra-articular MSCs. |
| Magri C et al.  Plos One 2019 [54] | 22 Horses | Metacarpo- / metatarso- phalangeal  joint | Natural occurring | Single MSC injection (n=10) Double MSC injections (n=12) | Umbilical cord  Allogeneic (Horse) | Expanded | 1 x 10^7^ cells | - | Single vs double injection 1 -month interval 2 ml | 6 months | There is no apparent clinical benefit of repeated intra-articular administration of MSCs in horses with OA when compared to the effect of a single injection. |
| Zhang BY et al. Stem Cells Int 2018 [106] | 8 Dogs | Knee | Cartilage drilling | MSC (n=4) OA control (n=4) | Umbilical cord  Allogeneic (Dog) | Expanded | 1 x 10^6^ | - | Single injection - 1 ml | 1 month | Canine MSCs promote the repair of cartilage and patella injury by OA, improve the surrounding injury tissue of the joints, and reduce the inflammation response. |
| Saulnier N et al. Osteoarthritis Cartilage 2015 [78] | 30 Rabbits | Knee | Medial Meniscus destabilization | Early injection MSC (n=14) Late injections MSC (n=7) OA control (n=9) | Umbilical cord  Xenogeneic (Horse) | Expanded | 3.5 x 10^6^ | - | Single injection - 150 μL | 2 months | Early MSC injection prevented OA progression and inhibited synovial inflammation. |
| Hsu CC et al. Int J Mol Sci 2020 [38] | 42 Rats | Knee | ACL transection + medial menscectomy | MSC ASC ESWT ESWT + MSC ESWT + ASC OA Control Sham control | Wharton Jelly  Xenogeneic (Human) | Expanded | 1 x 10^6^ | - | Single injection - 200 μL | 3 months | The use of Wharton Jelly MSC was effective, however the effects obtained were less than with ASC. |
| Cheng JH et al. Am J Transl Res 2019 [17] | 30 Rats | Knee | ACL transection + medial menscectomy | MSC + ESWT (n=6) MSC (n=6) ESWT (n=6) OA control (n=6) Sham control (n=6) | Wharton Jelly  Xenogeneic (Human) | Expanded | 1 x 10^6^ | - | Single injection 100 µl | 3 months | MSC treatment slowed OA progression, combined therapy with ESWT achieved lower Synovitis scores. |

ASA, amniotic suspension allograft; ASCs: adipose derived MSCs; BMMSCs, bone marrow derived MSCs; BMP-4, bone morphogenetic protein 4; CAM, cartilage acellular matrix; CM, conditioned medium; ESWT, Extracorporeal Shockwave therapy; FBS, fetal bovine serum; FGF18, fibroblast growth factor-18; GFP, green fluorescent protein; GM, granulocyte colony-stimulating factor mobilized; GO, Graphene Oxide; HA, hyaluronic acid; HPE, human placenta extract; IA, intra-articular; IDO1, indoleamine 2,3 dioxygenase 1; IL-10, interleukin 10; iNOS, Inducible nitric oxide synthaseIV, intra-venous; m-Dhacm, micronized dehydrated human amnion/chorion membrane; MSCs; MIA, mono-iodoacetate; MRI, magnetic resonance imaging; MSCs, mesenchymal stem/stromal cells; OA, osteoarthritis; PG, propylene glycol hydrogel; PRP, platelet rich plasma; RPS, reduced particle size; sFlt-1, Soluble vascular endothelial growth factor receptor-1; SVF, stromal vascular fraction
